# Two-stage CNNs for computerized BI-RADS categorization in breast ultrasound images

**DOI:** 10.1186/s12938-019-0626-5

**Published:** 2019-01-24

**Authors:** Yunzhi Huang, Luyi Han, Haoran Dou, Honghao Luo, Zhen Yuan, Qi Liu, Jiang Zhang, Guangfu Yin

**Affiliations:** 1 Department of Biomedical Engineering, College of Materials Science and Engineering, Sichuan University, Chengdu, 610065 China; 2 College of Electrical Engineering and Information Technology, Sichuan University, Chengdu, 610065 China; 3 National-Regional Key Technology Engineering Laboratory for Medical Ultrasound, Guangdong Key Laboratory for Biomedical Measurements and Ultrasound Imaging, School of Biomedical Engineering, Shenzhen University, Shenzhen, 518060 China; 4 Department of Ultrasound, West China Hospital of Sichuan University, Chengdu, 610041 China; 5 Bioimaging Core, Faculty of Health Sciences, University of Macau, Macau SAR, China

**Keywords:** Breast tumor in ultrasound image, Breast Imaging Reporting and Data System (BI-RADS), Automatic categorization, Deep convolutional neural network

## Abstract

**Background:**

Quantizing the Breast Imaging Reporting and Data System (BI-RADS) criteria into different categories with the single ultrasound modality has always been a challenge. To achieve this, we proposed a two-stage grading system to automatically evaluate breast tumors from ultrasound images into five categories based on convolutional neural networks (CNNs).

**Methods:**

This new developed automatic grading system was consisted of two stages, including the tumor identification and the tumor grading. The constructed network for tumor identification, denoted as ROI-CNN, can identify the region contained the tumor from the original breast ultrasound images. The following tumor categorization network, denoted as G-CNN, can generate effective features for differentiating the identified regions of interest (ROIs) into five categories: Category “3”, Category “4A”, Category “4B”, Category “4C”, and Category “5”. Particularly, to promote the predictions identified by the ROI-CNN better tailor to the tumor, refinement procedure based on Level-set was leveraged as a joint between the stage and grading stage.

**Results:**

We tested the proposed two-stage grading system against 2238 cases with breast tumors in ultrasound images. With the accuracy as an indicator, our automatic computerized evaluation for grading breast tumors exhibited a performance comparable to that of subjective categories determined by physicians. Experimental results show that our two-stage framework can achieve the accuracy of 0.998 on Category “3”, 0.940 on Category “4A”, 0.734 on Category “4B”, 0.922 on Category “4C”, and 0.876 on Category “5”.

**Conclusion:**

The proposed scheme can extract effective features from the breast ultrasound images for the final classification of breast tumors by decoupling the identification features and classification features with different CNNs. Besides, the proposed scheme can extend the diagnosing of breast tumors in ultrasound images to five sub-categories according to BI-RADS rather than merely distinguishing the breast tumor malignant from benign.

## Background

Breast cancer is the leading cause of morbidity and mortality in women worldwide [1–3]. To prevent needlessly biopsies and reduce unnecessary expenses and anxiety for thousands of women each year [4, 5], screening ultrasound is usually leveraged in most of the routine examination and clinical diagnosis [6–9]. Clinically, the Breast Imaging Reporting and Data System (BI-RADS) [10] provides a guidance and criteria for physicians to determine the categories of breast tumor based on medical images. According to BI-RADS assessments for ultrasound images (listed in Table 1) and the extensive clinical practice of our advisor committee members at the West China Hospital of Sichuan University in Chengdu, China, three categories, including Category 3, Category 4, and Category 5, are the major distribution during ultrasound assessments [10]. Category 1 and Category 2 assessments do not exhibit any evidence of malignancy on sonography, which are recommended for another routine age-appropriate screening. Category 6 refers to biopsy-driven results. Particularly, lesions of Category 4 have an extensive range of likelihood of malignancy, i.e., from 2 to 95%. In clinic, Category 4 is further divided into three subcategories, including 4A, 4B, and 4C. The patients and referring physicians can make an informed decision about management after a biopsy correlation. Considering the concerns of physicians, an automatic breast tumor grading scheme should at least cover an objective diagnosis from Category 3 to Category 5, including the subcategories of Category 4 (refer to Table 1). However, physicians’ final assessment on the lesion may be different according to the deliberation in BI-RADS. Therefore, an automatic categorization system can relieve the burden of the manual diagnosis and reduce the individual bias.

**Table 1:** BI-RADS assessment categories for breast ultrasound images

Categories	Diagnosis assessment	
Category 0	Incomplete and needs additional imaging evolution	
Category 1	Negative	
Category 2	Benign	
Category 3	Probably benign (< 2% probability of malignancy)	
Category 4	Suspicious	
	Category 4A: Low suspicion for malignancy (2% to 8% probability of malignancy)	
	Category 4B: Moderate suspicion for malignancy (9% to 49% probability of malignancy)	
	Category 4C: High suspicion for malignancy (50% to 95% probability of malignancy)	
Category 5	Highly suggestive of malignancy (> 95% probability)	
Category 6	Known biopsy-proven malignancy	

There were a lot of semi-automated breast tumor classification methodologies, which employed hand-engineered features to better correspond to the probability of malignancy [11, 12]. However, semi-automated methods cannot totally relieve the diagnosis burden of physicians in nature [13]. Besides, majority of previous studies focused primarily on classifying breast tumors into benign and malignant [14–17]. When extending the relationship between the features extracted from breast ultrasound (BUS) images and the corresponding probability of malignancy into more complex categories, physicians need to spend extra time and effort to provide specific and appropriate handcrafted features. Recently, through exploiting hierarchical feature representations automatically learned from large-scale dataset, deep learning techniques have successfully addressed numerous medical image analysis problems [18–24]. Due to the superiority of deep learning in automatic feature extraction, several related works on detecting breast tumors from US images utilized deep learning methods instead of traditional feature engineering [20, 21, 23–26]. For example, Yap et al. [25] attempted to detect breast ultrasound lesion with different convolutional neural network (CNN) models, including a Patch-based LeNet model, a U-Net model and a transfer learning approach with a pre-trained FCN-AlexNet. Bian et al. [26] developed the detection work on automated whole breast ultrasound (AWBU) with a deep convolutional encoder-decoder network. Generally, the detection of breast tumor can preliminarily provide region of interests (ROIs) for the successive tumor classification task. More effective tumor/lesion region can guide CNNs to learn better discriminative features for the classification task. A few studies have validated the feasibility of classifying breast tumor into different categories with CNNs [23, 27–29]. Huynh et al. [27] highlighted that deep learning can be a promising new direction for obtaining “good” features for automatic breast tumor classification by comparing the results with those of typical methodologies (quantized features + typical classifier). However, the detailed information about the breast tumor classification network was not provided. Zhang et al. [28] has demonstrated the feasibility of using a CNN in classifying breast tumors with shear wave elastography (SWE). Although features from SWE images are helpful in localizing breast tumors, equipping each ultrasound device with SWE is not practical. And the image features may involve an abundance of interference because the contour determined by SWE is rather coarse. Moreover, the attempt only focused on classifying the BUS images into benign or malignant. At present, few studies developed the research on the automatic multi-category classification based on the BI-RADS. He et al. [29] implemented the multi-category classification based on electronic medical records from the aspect of natural language description. Clinically, a direct analysis on the tumor’s category based on the collected BUS images can better assistant physicians in relieving the diagnose burden. Due to abundant noise and interference from other tissues in BUS images, it is a rather challenging task to implement accurate multi-category classification corresponding to the BI-RADS only with the BUS images.

In this paper, the systematic research on breast tumor grading is performed based on the CNN architecture. To effectively learn the discriminative features of more detailed categories from BUS images, two considerations are taken into our fully automated categorization system: (1) identifying the tumor region from BUS images to reduce the influence of other tissues, and (2) making full use of the features in BUS images to increase the discriminative ability. The contributions of this paper are elaborated as follows;

1. This is the first comprehensive quantized grading system depending on BUS images, which can achieve a 5-score categorization based on BI-RADS, covering Category 3, Category 4a, Category 4b, Category 4c, and Category 5, thus potentially relieving the burden of a tedious image review process and alleviating subjective influence due to physicians’ experiences in clinical practice.
2. With our two-stage CNNs, features can be decoupled in the detection phase and classification phase, since the weights of the identification task and classification task cannot be well compatible in a one-stage CNN architecture for BUS images. Our two-stage system can perform better accuracy than the state-of-art one-stage methods.

## Materials and methods

### Subjects

In this study, all the collected 2-D breast ultrasound (BUS) images were from female patients and contained a breast tumor. For each volunteer participant, only one case corresponding to the maximum cut surface of breast tumor was used to generate the datasets. This study included 531 cases of Category 3, 443 cases of Category 4A, 376 cases of Category 4B, 565 cases of Category 4C, and 323 cases of Category 5. Human subject ethical approval was obtained from a relevant committee at West China Hospital of Sichuan University before collecting ultrasound images. Each subject provided written consent prior to the research. Philips IU22 ultrasound scanner (Philips Medical System, Bothell, WA) with a 5- to 12-MHz linear probe was utilized while collecting the data.

### Method overview

The CNN architecture is an extensively utilized deep learning technique for analyzing medical images [18, 30]. Typically, a CNN is constructed with several convolution layers [31, 32], maxpooling layers [33], and fully connected layers [34]. And the extensively utilized activation methods in the CNN include the rectified linear unit (ReLU), sigmoid, and tanh [35].

**Fig. 1:**
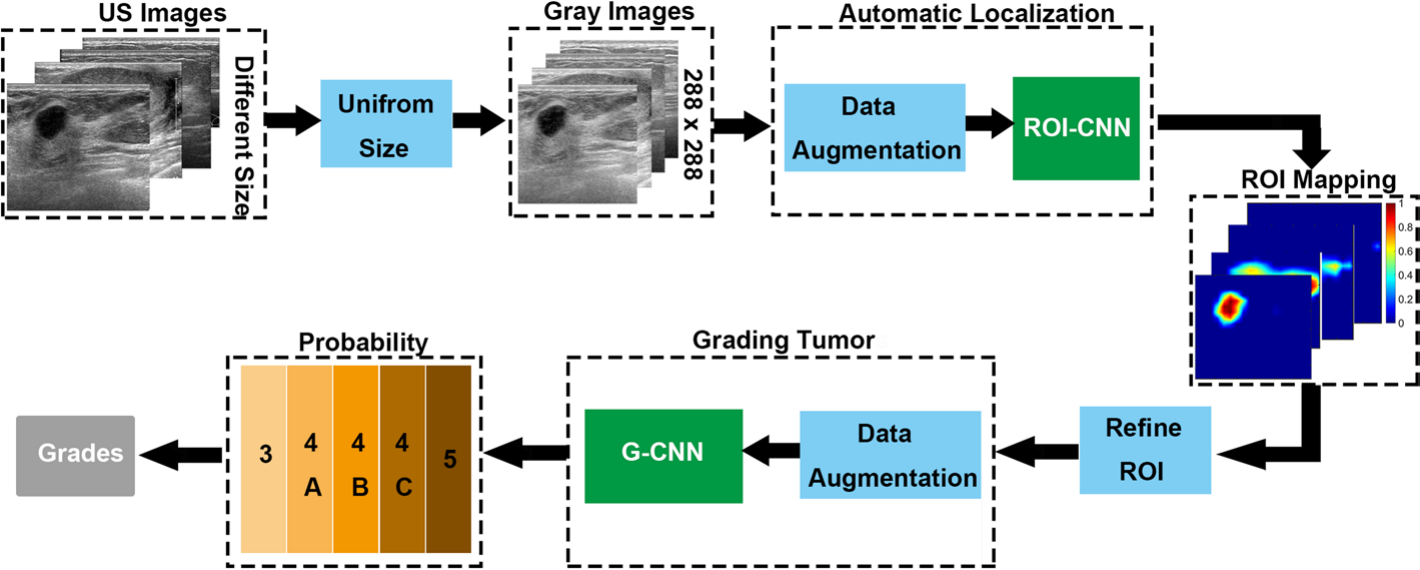
Schematic Illustration of our methodology. The input data was first unified into the same size 288*288. Then, the ROI-CNN identified the tumor region from the breast ultrasound image. The outputs of the ROI-CNN can be further improved by the refinement procedure. Finally, the G-CNN learned the differentiation of the input and rigorously classified the tumor into one of three categories (Category 3, Category 4, and Category 5), where Category 4 could be divided into three subcategories (Category 4A, Category 4B, and Category 4C)

Based on CNN architecture, the schematic illustration of our breast tumor categorization system is exhibited in Fig. 1. First, all input images were scaled into a uniform size of 288 × 288. Second, the ROI-CNN was designed to automatically identify the rough localization of the breast tumor Since the predicted ROIs of the ROI-CNN may mix other non-tumor regions and loss several important texture or boundary information, the following refinement procedure, including area filtering and the perfect Chan–Vese (C–V) level-sets methodology [36], was introduced to enable the identified ROI to be better tailored to the real boundary of the breast tumor. Finally, the G-CNN model was applied to analyze the refined ROIs by rating them with a score of five, as five categories of breast tumors were involved in the classification.

### CNN-based localization and grading models

Inherent speckle noise and low image contrast of the US images may bring unnecessary distraction while extracting features, thus making the automatic classification of the breast US images difficult. To extract effective features for the classification, the tumor identification network (ROI-CNN) and refinement procedure were first exposed on the whole BUS image to determine the effective ROI. Then, the following tumor grading network (G-CNN) can focus on extracting the discriminative features for classifying tumors.

#### The identification model—ROI-CNN

To effectively reduce the influence of other tissues, like Cooper’s ligaments, identifying the tumor from the corresponding whole BUS image is the first and most important procedure for implementing the automatic grading system. Our ROI identification network (ROI-CNN) was developed based on the fully convolutional networks (FCN) [37].

**Fig. 2:**
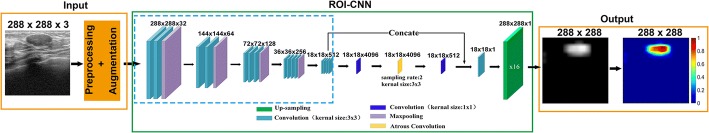
Illustration of breast tumor identification procedures. The input orange box denotes that the uniform-size images would first undergo the preprocessing and augmentation procedures. The green rectangle refers to the ROI-CNN architecture

Considering that the tumor size varies among different patients, the designed identification network requires to be robust and effective on the tumor with different size. To increase the feasibility of ROI-CNN on variable target size, our designed ROI-CNN introduced a multi-scale architecture based on the typical FCN-16s network. Firstly, a typical VGG network (refer to the blue dashed box in Fig. 2) was utilized to extract features. After four times down sampling, the output size of feature map from the VGG is 18 × 18, to further extract high level features, we need to compress the size of the feature maps.

However, too small size of the feature maps cannot well reflect the detailed boundary information of the breast tumor. In this study, a atrous convolution layer (refer to the yellow block in Fig. 2) was then incorporated into our ROI-CNN, which can effectively enlarge the receptive field of filters and capture a larger context without increasing the amount of parameters or the cost of computation [38–40]. In the atrous convolution layer, the kernel size was set to be 3 × 3 and the dilation rate was set to be 2. Besides, concatenating feature maps from different depths was performed (refer to Fig. 2) to ensure that features with two different receptive fields can be merged together. Following the atrous convolution layer, a convolution layer was additionally used as a transitional layer between the atrous convolution layer and the top convolution layer to provide balanced number of features from the deep layer and shallow layer for the concatenation operation. In the transitional convolution layer, the kernel size was set to 1 × 1 and the number of filters was set to 512. For the output of the ROI-CNN, the predicted identification possibility in the breast tumor region should be higher than the non-tumor region.

#### The grading model—G-CNN

Effective classifier can enhance the distinguishing ability of tumor features from different categories, thus promoting the accurate classification. Clinically, apart from the inner texture of breast tumors, the texture and the boundary information are also significant for classifying the breast tumors into different grades [10, 14]. Therefore, the grading model needs to take the texture and the boundary features into consideration to enhance the expression of the grading features.

**Fig. 3:**
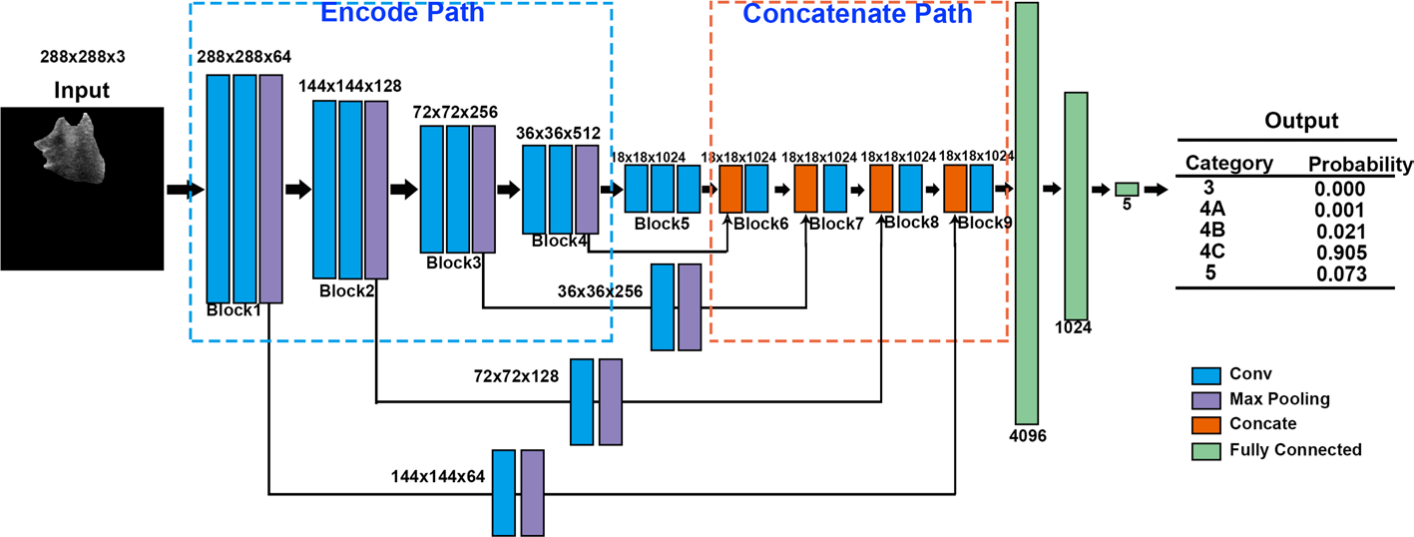
Illustration of the G-CNN network. The blue dashed rectangle box represents the encode path and the orange dashed rectangle box refers to the concatenate path. Different colors correspond to different operations. Each blue box corresponds to a convolution layer with a kernel size of 3 × 3, each purple box denotes a max pooling layer with a stride of 2, each orange box denotes as a concatenation layer, and each green box represents a fully connected layer

Usually, the texture and the boundary information are well represented in the low-level convolution layers, and the essential features can be well extracted with more convolution layers. In our proposed G-CNN, feature maps from different depths were concatenated together to make full use of the low-level and high-level information. Referring to Fig. 3, the G-CNN model was consisted of 9 blocks. The first four blocks (from Block 1 to Block 4) formed the encode path. Each block in the encode path shared the same structure, which contained two convolution layers and one max pooling layer. In the encode path, the number of feature channels of the convolution layer was doubled when followed by the max pooling layer. Following the encode path is the Block 5 contained of three convolution layers. The concatenate path was then followed with the four same blocks (from Block 6 to Block 9). Each block in the concatenate path was consisted of a concatenation layer and a convolution layer. In our G-CNN model, the feature maps from the lower layer was additionally concatenated with the feature maps from the deeper layer. Each block in the encode path was exploited to provide low-level features for the corresponding block in the concatenate path. For example, Block 3 and Block 7 were concatenated together. Note that, to ensure the size of the two inputs imported into the concatenation layer was consistent, the first three blocks in the encode path were followed by a convolution layer and a max pooling layer.

Totally, the G-CNN network contained 18 convolution layers. The batch normalization strategy [41] was encapsulated at the top convolution layer in each block, and the first two FC layers, to regularize the model. A L2 regularization operation was performed to reduce overfitting, which can enable better test performance via better generalization. The kernel size of all convolution layers was 3 × 3, and each layer was followed by ReLU [34]. All the max pooling layers was set to be 2 × 2 with a stride 2. At the end of the G-CNN were three fully connected (FC) layers that consisted of 4096 neurons, 1024 neurons and 5 neurons. A softmax layer followed the topmost FC layer with five neurons to conduct the grading output.

### The refinement

Affected by the ambulant speckle noise and other tissues in the BUS image, the prediction of the ROI-CNN may involve non-tumor region besides the tumor region. Moreover, the contour of the predicted region may have a bias from that of the real tumor contour. Therefore, additional refinement is imperative to ensure the effectiveness of the predicted ROI.

To ensure that only the lesion was export to subsequent grading system and improve the accuracy of the final categorization, the rough ROI from the ROI-CNN, which enclosed the breast tumor region, was then further refined by the following steps: (1) remove the connected domain with a smaller area (smaller than 40% of the max area) and choose the connected region closest to the image center; and (2) refine the boundary with a typical C–V level-sets methodology [36].

1 $$E\text{(}C\text{)} = \mu_{1} \int\limits_{inside(C)} {\left| {I(x,y) - c_{1} } \right|}^{2} dxdy + \mu_{2} \int\limits_{outside(C)} {\left| {I(x,y) - c_{2} } \right|}^{2} dxdy + \alpha \kappa$$

where $I$ is the image, $C$ refers to the boundary of the segmented region, $c_{\text{1}}$ and $c_{\text{2}}$ are the respective averages of $I$ inside and outside $C$, and $\kappa$ is the curvature of $C$.

#### Implementation details

- Loss function

In the ROI-CNN, the Dice loss function can be expressed as follows,

2 $$L_{ROI} = 1 - \frac{{2\left| {A_{PRED} } \right| \cdot \left| {A_{GT} } \right|}}{{\left| {A_{PRED} } \right| + \left| {A_{GT} } \right|}}$$

where *PRED* denotes the predicted ROI, and *GT* corresponds to the ground truth. *A*_*PRED*_ and *A*_*GT*_ refer to the predicted tumor area and the ground truth tumor area, respectively.

In the G-CNN, multi-class cross entropy [42] was employed as the loss function,

3 $$J(\theta ) = - \frac{1}{m}\left[ {\sum\limits_{i = 1}^{m} {y^{(i)} logf_{\vartheta } (I^{(i)} ) + (1 - y^{{^{(i)} }} )log(1 - f_{\vartheta } (I^{(i)} ))} } \right]$$

where *m* denotes the number of classes and *y* is the class label of each input. Both variables range from one to five. *ϑ* represents the parameters of the G-CNN, and *f*_*ϑ*_ corresponds to the mapping relationship from the input image *I* to the predicted output *f*_*ϑ*_(*I*).

In the G-CNN, each input can generate an output vector with size 1 × m, where the category with the highest possibility was taken as the predicted result.

b. Train process

Our proposed framework was implemented on Tensorflow and all experiments were conducted on a workstation equipped with a 2.40 GHz Intel Xeon E5-2630 CPU and an NVIDIA GF100GL Quadro 4000 GPU.

During the training phase of the ROI-CNN, the layers in the blue dotted box (refer to Fig. 2) were initialized with a VGG model [43] based on a pre-trained image classification dataset provided by ImageNet Large-Scale Visual Recognition Challenge in 2012 (ILSVRC-2012 CLS). The other layers in the ROI-CNN were initialized with a Gaussian randomizer. The minibatch size involved 16 images, and the optimizer SGD [44, 45] was set with a learning rate of 0.0001 and a momentum of 0.9 until convergence was attained.

In the training phase of the G-CNN, Random initialization was employed to yield better performance and faster convergence. 16 images were set as the minibatch size, and the SGD optimizer was set with learning rates of 0.001 which would be gradually decreased by a factor of 0.9 until convergence was attained.

### Performance evaluation

To validate the effectiveness of the grading scheme for breast tumors from US images, the localization and grading results were evaluated by comparing the corresponding manual annotations and labeling from the three physicians. The experiments implemented two aspects to assess our grading system. One was the effect of different options in tumor identification stage on the final grading results, and the other was the discriminative capability for different breast tumor categories.

#### The accuracy of the identified tumor

Three metrics were utilized to quantitatively evaluate the similarity between the predicted contour and the ground truth contour, including the Dice similarity coefficient (DSC) [46, 47], Hausdorff distance between two boundaries (HDist) [47, 48], and average distance between two boundaries (AvgDist) [47]. DSC was employed to examine the overlapping areas between the two comparisons. HDist and AvgDist were exploited to measure the Euclidean distance between a computer-identified tumor boundary and the boundary determined by physicians. Higher DSC, lower HDist, and lower AvgDist corresponded to more similarity between the two boundaries. Furthermore, AUC values and ROC curves were exploited to evaluate the performance of different experiments with a variable scope of ROIs.

### Experiment configurations

#### Image data involved in each stage of the categorization system

The input size of our two-stage grading system was set to 288 × 288. And fivefold cross-validation was employed to construct the training and testing datasets.

- *Image annotation*
  Each involved image was scored by three physicians with more than 3 years of experience performing BUS examinations based on the BI-RADS criteria. If the physicians differed in their annotations of the category, they discussed and then made consensus on the final category of the breast tumor.
- *Data preprocessing and augmentation*
  Due to the sample size of volunteer patients is limited, effective data preprocessing and augmentation is imperative for medical image datasets. The premise of augmentation is that the ROI must be incorporated into all augmented data regardless of the type of transformation exposure on the dataset.

1. *Data augmentation in the ROI-CNN model*
  In the ROI-CNN training stage, the augmentation times of each input were set to the same with the number of training epochs. This type of augmentation can enhance the randomization of input data and reduce the possibility of overfitting of the trained model, thus improving the robustness of the ROI-CNN model. Each input image was followed by the subsequent procedures in each calculated epoch, including random brightness, random contrast, random movement, random flip, and standardization. Each input can export *N* times outputs while experiencing *N* epochs. Conversely, in the testing phase, only standardization was exposed using input samples.
2. *Data augmentation in the G-CNN model*
  In the G-CNN training stage, to maintain the shape textures of breast tumors for the final classification, only geometric translation and flipping were involved. The original datasets were augmented four times with random movement, in which two augmentations were followed by horizontal flipping.

#### Effect of identification accuracy on the final grading

The coverage of localization, which denotes the area of the ROI, theoretically affects the feature mapping and may influence the final grading. To investigate the effects of the accuracy of the identified breast tumor on the final categorization from the BUS images, three types of import into the G-CNN with the corresponding experiments were involved and denoted as “No ROI-CNN”, “No Refined ROI-CNN”, and “Refined ROI-CNN”. “No ROI-CNN” corresponded to the experiment in which the input to the G-CNN directly applied the C–V level-sets method to input US images and lacked the prediction on the rough localization by the ROI-CNN. In the “No Refined ROI-CNN” experiment, the output of the ROI-CNN was not refined and was directly exported to the G-CNN. In the “Refined ROI-CNN” experiment, the original US images underwent complete processing procedures in our designed scheme.

The parameters of our designed method, experiment “Refined ROI-CNN”, were set as follows; (1) *μ*_1_, *μ*_2_, and *α* in equation (4) were all set to 1; (2) the maximum number of contour evolution iterations was set to 50. The parameters *μ*_1_, *μ*_2_, and *α* in the C–V level-sets experiment was the same as those in our refined ROI experiment. But the maximum number of contour evolution iterations in the “No ROI-CNN” experiment was set to 1000.

#### One-stage vs. two-stage categorization of BUS images

Making full use of the effective features is likely to achieve better categorization of breast tumor. To investigate the superiority of our two-stage system on grading BUS images, the accuracy of the predicted categorization of tumor was employed as an indicator, we compared the categorization of two-stage grading system with that of the one-stage grading architecture, which directly classified input breast US images into six classes, including the background and five breast tumor categories.

There are two types of the two-stage methods, one is with the refinement procedure, and the other is without the refinement procedure. In each type of the two-stage methods, we compared our G-CNN model with the other two typical classification network, one is the VGG network [43], and the other is the ResNet50 network [49]. Totally, there are six experiments in the two-stage methods. For the one-stage classification methods, three experiments are included: (1) experiment “One-stage G-CNN”, which directly classified the input into 5 categories with the our proposed G-CNN architecture (refer to Fig. 3); (2) experiment “One-stage VGG”, which directly classified the input BUS image into 5 categories with the VGG architecture; (3) experiment “One-stage ResNet”, which directly classified the input BUS image into 5 categories with the ResNet50 architecture.

## Results

### Effect of the identification on final grading accuracy

With DSC, AvgDist, and HDist, Table 2 exhibits the comparison on the similarity of the generated tumor areas from experimental cases “No ROI-CNN”, “No Refined ROI-CNN”, and “Refined ROI-CNN”. From Table 2, we can observe that the ROI from the experimental case “Refined ROI-CNN” can perform the highest DSC average value and lowest AvgDist average and HDist average, thus enabling the most similarity to the real tumor region of the three listed experiments. Compared with “Refined ROI-CNN”, the experiment “No Refined ROI-CNN” lacks the refinement procedure to further determine the ROI from BUS images. With lower DSC average, higher AvgDist average, and higher HDist average, the similarity of the predicted ROI from the experiment “No Refined ROI-CNN” is less than that of the experiment “Refined ROI-CNN”. Compared with the CNN-based experimental test cases, experiment “No ROI-CNN” directly identifies the tumor with level-sets, which illustrates the worst performance on the average values of DSC, AvgDist, and HDist. This is because image contrast, speckle noise in BUS images, and initial contour, are likely to affect the evolution procedure of C–V level-set, thus locating an erroneous target and resulting in undesirable performance on the predicted contour. Therefore, referring to Table 2, the similarity between the predicted ROI and the ground truth manually outlined by the three physicians can gradually be increased with every improvement of implementation from the experimental test cases.

**Table 2:** Comparisons of different identification implementations

	DSC	AvgDist (pixel)	HDist (pixel)
No ROI-CNN	0.6007 ± 0.0302	51.2115 ± 2688.8	82.7174 ± 3423.1
No-Refined ROI-CNN	0.8665 ± 0.0133	7.6764 ± 157.8465	23.5292 ± 531.9632
Refined ROI-CNN	*0.9125* ± 0.0015	*3.9668* ± 7.0654	*11.0110* ± 40.6948

Experiments “Refined ROI-CNN”, “No-Refined ROI”, and “No ROI-CNN” were compared with three similarity measurements, including DSC, AvgDist, and HDist

The significance for the italic values is to illustrate the method with the best performance according to each evaluated metric

**Fig. 4:**
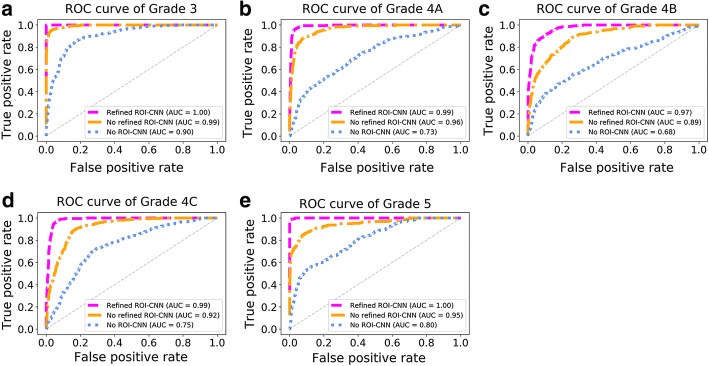
ROC curve and AUC values of different implementations in grading breast tumors. The implementations encompassed “Refined ROI-CNN”, “No refined ROI-CNN”, and “No ROI-CNN”. **a**–**e** Correspond to the results of Category “3”, “4A”, “4B”, “4C”, and “5”

Furthermore, Fig. 4 exhibits the ROC curves in the grading categories based on different predicted ROIs resulted from the experiments “No ROI-CNN”, “No Refined ROI-CNN”, and “Refined ROI-CNN”. Referring to the ROC curves of each category, the best grading results can be achieved with the experiment “Refined ROI-CNN” with the highest AUC. In contrast, experiments “No Refined ROI-CNN” and “No ROI-CNN” provide the G-CNN with ROIs of less quality, thus the AUCs are lower. Particularly, the lower quality of the ROI, the lower AUC is referring to Fig. 4, “No ROI-CNN” performs the worst the AUC in all the experiments by providing the lowest similarity of the ROI (see in Table 2). Therefore, based on comparisons of DSC, AvgDist, HDist, and ROC curves, the best performance for localizing ROI can be achieved with the “Refined ROI-CNN”.

### One-stage vs. two-stage framework

The grading accuracy for each tumor category of the one-stage and the two-stage experiments is listed in Table 3. The one-stage methods refer to directly predicting the unified image (288*288) into five categories without the identifying procedure. Experiment cases of the one-stage methods are consisted of “One-stage G-CNN”, “One-stage VGG”, “One-stage ResNet”. The two-stage methods mean that an extra identification procedure is involved to facilitate the implementation of the classification task. There are two types of two-stage methods, one is the with the refinement procedure and the other is without the refinement procedure. The two-stage methods with refinement procedure are consisted of experiment case “Refined ROI-CNN + G-CNN”, experiment case “Refined ROI-CNN + VGG”, and experiment case “Refined ROI-CNN + ResNet”. The two-stage methods without refinement procedure involve the following experiment cases: “ROI-CNN + G-CNN”, “ROI-CNN + VGG”, and “ROI-CNN + ResNet”.

**Table 3:** Comparisons of one-stage models and two-stage models with the grading accuracy of each category

	Category 3	Category 4A	Category 4B	Category 4C	Category 5
Refined ROI-CNN + G-CNN	*0.998* ± *0.0040*	*0.940* ± *0.0110*	*0.734* ± *0.0662*	*0.922* ± *0.0376*	*0.876* ± *0.1234*
Refined ROI-CNN + VGG	0.990 ± 0.0047	0.920 ± 0.0194	0.673 ± 0.0692.	0.908 ± 0.0493	0.841 ± 0.1319
Refined ROI-CNN + ResNet	0.991 ± 0.0052	0.927 ± 0.0155	0.688 ± 0.0665	0.920 ± 0.0388	0.858 ± 0.1423
ROI-CNN + G-CNN	0.955 ± 0.0076	0.897 ± 0.0112	0.679 ± 0.0678	0.906 ± 0.0434	0.837 ± 0.1232
ROI-CNN + VGG	0.947 ± 0.0145	0.864 ± 0.0132	0.667 ± 0.0726	0.865 ± 0.0463	0.818 ± 0.1281
ROI-CNN + ResNet	0.954 ± 0.0136	0.878 ± 0.0124	0.669 ± 0.0664	0.899 ± 0.0425	0.835 ± 0.1338
One-stage G-CNN	0.797 ± 0.0190	0.552 ± 0.0312	0.496 ± 0.0876	0.715 ± 0.0674	0.559 ± 0.1257
One-stage VGG	0.723 ± 0.0244	0.533 ± 0.0471	0.460 ± 0.0888	0.644 ± 0.0535	0.436 ± 0.1328
One-stage ResNet	0.755 ± 0.0268	0.550 ± 0.0302	0.472 ± 0.0799	0.692 ± 0.0585	0.508 ± 0.1402

One-stage models were consisted of experimental test cases “One-stage G-CNN”, “One-stage VGG” and “One-stage ResNet”. Two-stage models involved six experiment test cases, including “Refined ROI-CNN + G-CNN”, “Refined ROI-CNN + VGG”, “Refined ROI-CNN + ResNet”, “ROI-CNN + G-CNN”, “ROI-CNN + VGG”, and “ROI-CNN + ResNet”

The significance for the italic values is to illustrate the method with the best performance according to each evaluated metric

Referring to Table 3, the one-stage with image-level classification methods (“One-stage G-CNN”, “One-stage VGG”, and “One-stage ResNet”) performs the worst on the average grading accuracy. Specially, the predicted accuracy of these methods can only achieve about 0.5 on the “Category 4A”, “Category 4B”, and “Category 5”. By introducing an extra identification network, the two-stage methods without refinement (“ROI-CNN + G-CNN”, “ROI-CNN + VGG”, and “ROI-CNN + ResNet”) can have an accuracy improvement on each category compared with the one-stage methods. Particularly, expect the “Category 4B”, the average accuracy of the other four categories are over 0.8 for the two-stage methods without refinement. Among of the listed three experiments (“ROI-CNN + G-CNN”, “ROI-CNN + VGG”, and “ROI-CNN + ResNet”), the experiment “ROI-CNN + G-CNN” achieves the highest accuracy on all the categories. And the experiment “ROI-CNN + VGG” performs the lowest accuracy among of the three methods. By successively adding an extra refinement procedure, we can observe that, the grading accuracy in each category of the two-stage methods with refinement procedure (“Refined ROI-CNN + G-CNN”, “Refined ROI-CNN + VGG”, “Refined ROI-CNN + ResNet”) becomes higher than that of the rest of the methods listed in Table 3. Particularly, in contrast to the typical classification models (VGG [43] and ResNet50 [49]), the methods with our G-NN network still performs better on the grading accuracy.

For the experiment “Refined ROI-CNN + G-CNN”, the predicted accuracy in Category “3” can reach an average of 0.998, which is rather close to one. Both Category “4A” and Category “4C” can achieve an average accuracy greater than 0.9, and the prediction accuracy for Category “5” can obtain an average of 0.876, which close to 0.9. Thus, we suggest that our predictions for the four categories are effective and highly accurate. Note that, the average accuracy of the Category “5” is less than 0.9, and the biased predictions for the Category “5” are primarily located in Category “4C”, due to the following two factors: 1) the number of samples in Category “5” is smaller than the numbers in the other categories, and 2) the ratio of the benign samples in Category “5” is higher than the ratio of the malignant samples in Category “5”. Although the prediction for Category “4B” cannot approach 0.8, it is higher than the statistical probablity. According to the criteria in Table 1, for each test tumor belonging to the Category “4B”, the probability of benign and malignant is rather close, even the experienced physicians can hardly determine the tumor’s category merely from the US image. Clinically, extra diagnostic tests such as biopsy, is required to determine the final accurate results. With only one type of source associated with US images, our two-stage grading system in Fig. 1 can achieve the best performance on grading accuracy than the other methods listed in Table 3.

### The grading accuracy predicted by our two-stage categorization system

**Fig. 5:**
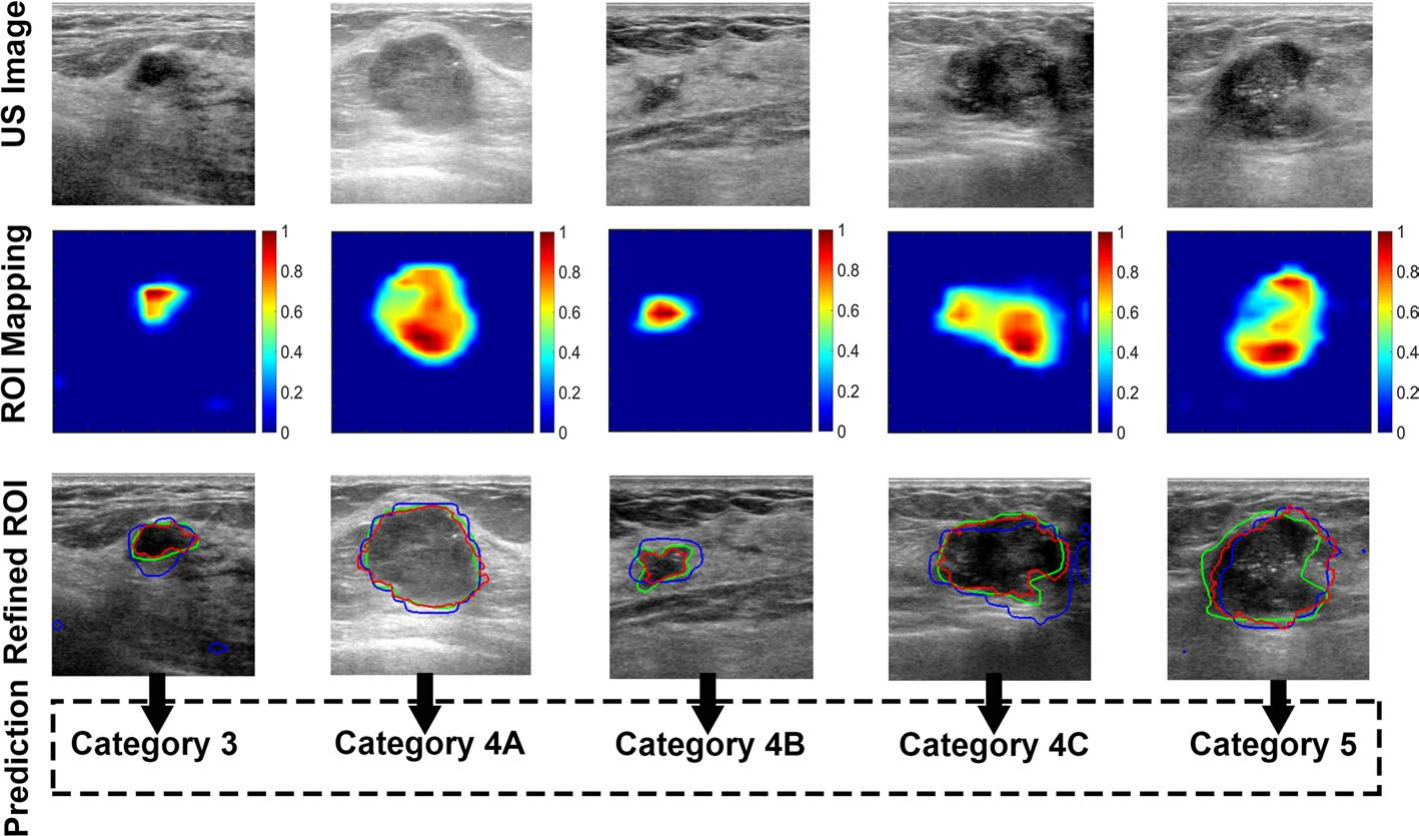
Intermediate and the end results of our grading scheme. The first row refers to the ultrasound images with uniform size. According to the test images in the first row, ROI-CNN predicts the possible rough ROI, which is shown in the second row. The third row illustrates the refined ROI based on the output of the ROI-CNN, where the green curves correspond to the contours determined by the three physicians, the blue curves denote the contours from the ROI-CNN output, and the red curves represent the refined contours based on the blue curves. The last row shows the predictions of the G-CNN with the refined ROI

Figure 5 sequentially illustrates the crucial intermediate results and the final predictions of the proposed methodology in Fig. 1 for several typical cases. The first row contains BUS images with a uniform size. The localization results of the ROI-CNN are presented in the second row in Fig. 5. We discovered that the ROI-CNN can effectively recognize tumors in BUS images. The ROI mapping results are depicted in jet colormaps, where warm color tones indicate a high possibility of tumor prediction and vice versa. The region with the highest possibility appears in the real tumor region (red part in figures). The centric possibilities of the other regions predicted by the ROI-CNN, are much lower than the centric possibility of the actual tumor. Another observation is that the mapping results can adequately reflect the approximate areas of tumors in the US images; however, the contours may not be smooth and located that close to the corresponding tumors. The third row in Fig. 5 represents the results from sequential refinement procedures on ROIs. The green curves represent the ground truth with agreement among the three physicians. The blue curves and red curves represent contours from the ROI-CNN and refinement procedure, respectively. We can observe that the refinement procedure can slightly improve the contour fitness of the ROI to the real tumor. With a suitable and effective ROI exported to the G-CNN, desirable grading result can be achieved. The results in the first column of the third row illustrate that refinement is a robust tool for eliminating the disturbance from other tissues.

## Discussion

Automatic quantitation of the category of breast tumor from US scanning can assist physicians in the tedious diagnosing task. This is the first comprehensive quantized grading evaluation on breast ultrasound images based on the criteria of BI-RADS. With the CNN architecture, which can automatically learn and extract goal-oriented features from images, our two-stage grading system can accurately identify the tumor region and discriminate the category of the breast tumor in ultrasound images. Our grading system can achieve a 5-score categorization of BI-RADS, covering Category 3, Category 4a, Category 4b, Category 4c, and Category 5, thus potentially relieving the burden of a time-consuming image review process and alleviating influence due to physicians’ experiences in clinical practice. Additionally, the proposed categorization system can ensure the robustness and effectiveness of the fully automated categorization system by decoupling the identification features and classification features.

The proposed two-stage architecture can make full use of the effective features from breast US images by effectively decoupling the information of identification and categorization, thus improving the final grading accuracy. The identification task focuses on distinguishing the tumor from the background, while the grading task concentrates on classifying the breast tumors into different classes, so the features used to identify tumor from the background are different from those applied to classify the tumor into five categories. Referring to Table 3, the accuracy of the final diagnosis illustrates that our two-stage CNNs can achieve better performance than the one-stage methods. The results in Table 3 indicates that the two-stage architecture is more suitable for grading BUS images, because the features of the identification task and categorization task cannot be well compatible. Therefore, a two-stage grading system can ensure higher accuracy, which is a rather vital indicator of medical image analysis, in classifying breast tumor categories.

In the two-stage grading system, the designed identification model and refinement procedure contribute to achieving an effective ROI for the following classification model, thus reaching a desirable grading result. Generally, additional irrelevant information imported to the G-CNN models may be translated into interference and produce an unsatisfactory grading result. The results in Table 2 and Fig. 4 also suggest that with more accurate and precise ROI input to the G-CNN, the better implementations for grading breast tumors are possible. Affected by the abundant speckle noise in ultrasound images, the contours resulted from the level-set methods [36] may occur large bias during the evolution process. Therefore, the single refinement procedure cannot generate effective ROIs for the following G-CNN model (refer to Table 2). Meanwhile, the predictions from the ROI-CNN are usually smooth in the boundary, some detail information will be lost particularly for the malignant cases, so the single ROI-CNN model is inadequate in providing a desirable ROI for the following classification network. Therefore, by combining the ROI-CNN model and the refinement procedure together, the predicted ROI can be closer to the real breast tumor so as to provide more elaborate ROIs for the following symmetric architecture G-CNN.

Table 3 illustrates that our G-CNN model performs better accuracy than the typical VGG [43] and RestNet 50 [49]. Generally, the enhancement of effective features can facilitate the final grading accuracy for the classification model. In our proposed G-CNN, the layers embedded in the concatenate path and the skip connections (refer to Fig. 3) can combine the lower dimensional feature maps and the higher dimensional features together, thus promoting discriminability for classifying different breast tumor categories. In contrast, the typical classification models, such as VGG [43] and RestNet 50 [49], only involve the encode path in Fig. 3 and export the high-level information to implement classification. However, the high-level features may suffer a loss of the texture or boundary information contained in the lower convolution layers. The texture and the boundary information usually provide important hints for the classification task according to BI-RADS. Due to the lack of a compensation strategy, ResNet50 and VGG cannot perform desirable accuracy on classifying the breast ultrasound images. Therefore, we conclude that better grading results can be achieved with the enhanced feature maps from G-CNN.

Our grading system has a desirable performance on the “Category 3”, “Category 4A” and “Category 4C”, which can obtain the accuracy greater than 0.9. But for the “Category 4B” and the “Category 5”, the grading accuracy of are lower than that of the other three categories (refer to Table 3). This may be caused by that the data amount of the “Category 4B” and “Category 5” are less than that of the other three categories. Although the prediction for Category “4B” cannot approach 0.8, it is significantly higher than manual decision. According to the criteria in Table 1, each tumor falling into the Category “4B” may have a close probability of being benign or malignant. Even the experienced physicians may have biases in determining the category only from the US image. Clinically, further diagnostic tests such as biopsy, is needed to achieve the final accurate results. With only one type of source associated with US images, our grading scheme can adequately predict the category of breast tumors. In the future, we will continue to collect more data, particularly on the “Category 4B” and “Category 5”, to further increase the prediction accuracy of our grading system. Moreover, we plan to integrate the images in Category 0, 1, 2, and 6 into current grading system, thus developing a comprehensive and complete computerized BI-RADs grading system.

## Conclusion

In this study, we proposed a two-stage automatic categorization system to quantize the criteria of BI-RADS and offer an objective assessment. Based on deep learning techniques, a series of comprehensive explorations were conducted using a combination of the procedures of CNN-based methods, typical image processing schemes, and the CNN architecture applicable to breast US images. The proposed scheme can also serve as an assistant computerized toolkit for the education of radiology residents and medical students to improve their discriminative skills in breast tumor examination with US scanning. Meanwhile, the proposed grading scheme based on CNN can be easily extended to analyses of other breast ultrasound images generated from other equipment without extra feature engineering.
